# Burnout and health status differences among primary healthcare professionals in Portugal

**DOI:** 10.1186/s12875-021-01425-9

**Published:** 2021-04-28

**Authors:** Pedro L. Ferreira, Vitor Raposo, Aida Isabel Tavares, Ana Pinto

**Affiliations:** 1 CEISUC - Centre for Health Studies and Research, University of Coimbra, Coimbra, Portugal; 2 FEUC - Faculty of Economics, University of Coimbra, Coimbra, Portugal; 3 ISEG -UL - Lisbon School of Economics and Management, University of Lisbon, Lisbon, Portugal; 4 FCTUC - Faculty of Science and Technology, University of Coimbra, Coimbra, Portugal

**Keywords:** Self-assessed health, Burnout, Well-being, Primary care, Portugal

## Abstract

**Background:**

This paper is focused on two indicators which may be considered as proxies of individuals’ well-being: self-assessed health and burnout intensity. There is little research relating these concepts with the type of the primary healthcare setting, its urbanization density and the region. The aims of this work are threefold: (i) to find determinant factors of individual health status and burnout, (ii) to find possible differences across different types of health care units, differently urbanized areas, and different administrative regions, and (iii) to verify if there are differences in between GPs and nurses.

**Methods:**

Data was gathered from an online questionnaire implemented on primary health care. A sample of 9,094 professionals from all 1,212 primary health care settings in Portugal mainland was obtained from an online questionnaire filled from January and April 2018. Statistical analyses include the estimation of two ordered probits, one explaining self-assessed health and the other the burnout.

**Results:**

The individual drivers for good health and lower levels of burnout, that is, better well-being, are estimated for GPs and nurses. Main findings support that, first, nurses report worst health than GPs, but the latter tend to suffer higher levels of burnout, and also that, 'place' effects arising from the health unit settings and regional location are more significant in GPs than in nurses. However, urbanization density is not significantly associated with health or burnout.

**Conclusions:**

A set of policy recommendations are suggested to improve the healthcare workforce well-being, such as improving job satisfaction and income. These policies should be taken at the health care unit level and at the regional administrative level.

## Background

Naturally, the major goal of any health system is to improve people's health [1]. To achieve this goal, the system should rely on a health care workforce to sustain its functioning. A motivated, productive, caring, and efficient workforce ensures that health care services are delivered in the most appropriate way to people, considering their needs and expectations [2]. In this way well-being is considered a key element to ensure that healthcare organizations meet the major health systems goals, e.g. health, responsiveness and protection for social and financial problems. Healthcare professionals show consistently across different countries high rates of sickness absence, burnout, and distress compared to other sectors [3].

There is evidence that physicians and nurses experiencing burnout are more likely to make poor decisions and mistakes, to display less empathic or even hostile attitudes towards patients and among colleagues. Healthcare professionals suffering from burnout are more willing to change jobs, report job dissatisfaction, and provide low quality care [4–8]. So there is an increasing concern to improve the wellbeing, the mental and the physical health, of health professionals [3, 9].

In our analysis, we focused our attention on two indicators, namely, self-assessed health and burnout which may be considered as proxies for individuals’ well-being. We take well-being as an umbrella and wide concept that accounts for good physical and mental health and lower burnout intensity. These two indicators cause opposite feelings in a person's well-being, by using the rationale that a good health contributes to a pleasant feeling, while burnout causes an unpleasant effect [10] (APA, 2020). In this way, a good perception of self-health is associated with a good level of well-being [11–13], while a high level of burnout is associated with a low level of well-being [14–16].

Well-being is a state of good health, happiness, fulfilment and positive perception of one’s life [17]. It is also considered as a multidimensional construct [18], accounting for several dimensions including mental, social, physical and spiritual well-being [19].

Burnout, on its turn, is a psychological, emotional and mental feeling of fatigue, exhaustion, helplessness and prostration. It has been defined in the 11^th^ Revision of the International Classification of Diseases (ICD-11) [20] as an occupational syndrome and not a medical condition. Feelings of low energy, exhaustion and fatigue are part of specific domains or spheres in the person’s life, including personal, work-related burnout, and client-related burnout [21].

The set of factors determining burnout is similar to the one that determines health [22, 23], including individual socioeconomic factors such as age, gender, education, marital status, and professional status. Concerning the determinants of burnout, the importance of occupational and organizational factors should also be stressed [24], as well as the factors related to job and career satisfaction [25, 26]. Empirical evidence reveals that lower levels of job and career satisfaction correlate with higher levels of burnout.

The 'place' where individuals work also influences health and burnout. On the one hand, regarding the relationship between ‘place’ and health, it is known that regional factors and urban density affect individual's health and interact with individual and socioeconomic factors [27–33]. On the other hand, less is known about the correlation between the urbanization density and region where individuals work and their burnout intensity.

Some studies show that physicians in urban hospitals tend to suffer more from burnout than those in smaller rural hospitals [34], and physicians in rural areas tend to report good job satisfaction due to low emotional exhaustion [35]. However, some authors [8] suggested that physicians and nurses in rural areas could suffer from higher levels of burnout, while others [36] did not find differences of burnout between rural and urban nursing units.

Empirical evidence reached different findings concerning the levels of burnout among physicians and nurses. Some studies place higher burnout on physicians [37], others on nurses [38], and others find no significant difference [39]. Professionals working in primary healthcare are more likely to suffer higher burnout than those working in a hospital [38], and prevalence of burnout among GPs is very high [25, 40], but burnout affecting physicians seems also to vary according to their specialty [41].

Little research exists in primary healthcare relating health status and burnout of health professionals with the type of setting where the care is provided, which includes the type of health unit, the urbanization and the region of location.

This work aims to contribute to this research field. Its purposes are of threefold. First, to estimate the determinant factors of individual well-being, measured by self-assessed health and by burnout level. Second, to find possible differences of well-being across different types of health care units, differently urbanized areas, and various administrative regions. In other words, this work aims to identify effects related to health care unit, urbanization and region on the well-being of GPs and nurses. And, finally, to verify if there are differences in health and burnout between GPs and nurses.

### Short description of the primary healthcare system in Portugal

In Portugal, the primary healthcare system has a relevant role in the overall health system as it works as a gatekeeper for hospital care. An international comparison of the importance of primary health care in Europe was presented by Kringos et al. [42]. They showed that Portugal as Belgium, Denmark, Finland, Spain and UK have a relatively strong primary care.

**Fig. 1:**
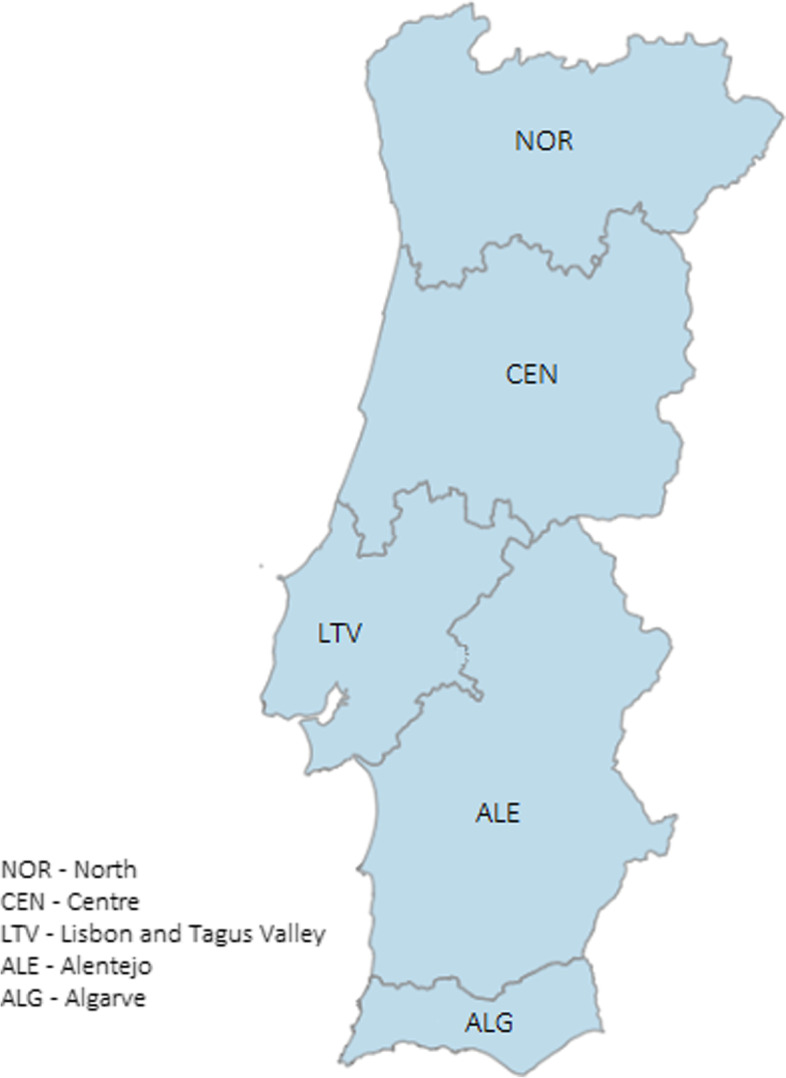
Regions in Portugal Mainland

Portugal mainland comprises five Regional Health Authorities: North (NOR), Central (CEN), Lisbon and Tagus Valley (LTV), Alentejo (ALE), and Algarve (ALG) (as shown in Figure 1 in Appendix). Each of these Regional Health Authorities is responsible for running several Groups of Primary Care Centers, which in turn include several primary health care units [43, 44].

These primary health care units may have different organization formats. In our work, we identify the so-called family health units (USF) and the traditional primary health care units (UCSP). The former can be classified in models A and B, based on the way they are organized and on the type of incentives paid to professionals. USF units have some level of autonomy and participative management. Professionals in USF-B receive a monetary incentive for performance, in particular, GPs, whereas in USF-A payments are only salaries.

On the other hand, UCSP have well established vertical hierarchies and very low level of autonomy. Other primary health care units include USP (public health unit), UCC (continued care unit), and URAP (shared assistance resources unit) [44, 45].

It is noteworthy that the access by users to primary healthcare services may be different across regions. These differences have been described in a previous work by Ferreira et al. [46]. For instance, in Lisbon and Tagus Valley region is more likely to find people who are not enrolled in a GP which makes access to medical appointments very difficult.

## Methods

### Study design and sampling

Data was gathered from an online questionnaire to health professionals, which includes GP, nurses, technicians and other health care professionals. The survey was applied to all 19,563 healthcare professionals in all 1,212 primary healthcare units in Portugal mainland, between January and April 2018. The questionnaire was answered in a private access e-platform (LimeSurvey), using a password system to ensure anonymity, non-duplication and answers only from the target population.

The questionnaire was approved by the Portuguese Authority for Individual Data Protection. It included three main groups of questions: (i) sociodemographic and labour characteristics, (ii) job satisfaction, and (iii) burnout.

Sociodemographic data included gender, age, education level and family status. Labour characteristics encompassed professional category, regular working hours, experience in coordinating, managerial or leadership functions, and how long the professionals have been working in the unit and in the profession. The questionnaire was designed to evaluate professionals’ satisfaction with their job and workplace.

Measurement of job satisfaction was based on the questionnaire ‘Hospital Employee Judgment System’ [47] and developed and validated to the Portuguese context by the Centre for Health Studies and Research of the University of Coimbra, Portugal [48]. The instrument measures the dimensions 'quality of the workplace', 'quality of services provided' and 'continuous quality improvement'. An overall level of satisfaction regarding the unit where individuals work was computed by weighted average based on those dimensions.

Professionals' burnout was measured by the Portuguese validated version of the Copenhagen Burnout Inventory (CBI) [21] that encompasses three scales of burnout: personal, work-related, and patient-related [49, 50]. According to CBI authors, personal burnout measures professional's perception of psychological fatigue and exhaustion. Work-related burnout refers to the extent to which a professional attaches his/her perception of physical and emotional fatigue and exhaustion to his/her work. And patient-related burnout measures how much s/he attributes his/her perceived feelings of physical and emotional fatigue and exhaustion to the work with patients. The overall burnout indicator is obtained by computing a simple average between those three dimensions of burnout.

### Variables

#### Dependent variables

The dependent variables used to capture professional well-being were self-assessed health (SAH) and burnout level, as summarized in Table 1. Self-assessed health was obtained from the question "how do you consider your health in general?" with the possible five answers, varying from 'excellent' to 'very poor.

**Table 1:** Dependent variables description

Group	Variable	Description
Well-being	burnout	Overall burnout indicator is a continuous variable, ranging from 0 (no burnout) to 100 (complete exhaustion)
	self-assessed health	Level of self-assessed health in categories, ranging from 1 (very poor) to 5 (excellent)

#### Independent variables

The set of independent variables include socioeconomic variables, professional and labour variables, satisfaction indicator, geographic indicators and unit type. These variables are described in Table 2.

**Table 2:** Independent variables description

Group	Variable	Description
Socio-economic	male	Dummy variable: 1 if male; 0 otherwise
	age	Number of years old
	married	Dummy variable: 1 if married or partnership; 0 otherwise
	nb_children	Number of children
	education	Years of completed level of education. It ranges from 4 years of primary school to 22 years of doctorate
	income	Family income sufficiency for family needs and personal training. Dummy variable: 1 if income is sufficient; 0 otherwise
Professional and labour	GP	Dummy variable: 1 if GP; 0 otherwise
	nurse	Dummy variable: 1 if nurse; 0 otherwise
	technician	Dummy variable: 1 if health technician; 0 otherwise Reference category for professional category
	experience	Number of years of professional experience
	tenure	Dummy variable: 1 if permanent and tenure contract; 0 otherwise
Job Satisfaction	Overall job satisfaction (OJS)	Level of overall professional satisfaction, ranging from 0 (completely unsatisfied) to 1 (completely satisfied)
Geographic	urban	Dummy variable: 1 if geographical area with more than 5,000 inhabitants and a population density higher than 500 inhabitants per Km^2^; 0 otherwise
	rural	Dummy variable: 1 if area with less than 2,000 inhabitants and a population density lower than 100 inhabitants per Km^2^; 0 otherwise
	moderately urban	It is defined in-between urban and rural areas defined above. Reference category for urbanization density
	NOR	Dummy variable: 1 if Northern region; 0 otherwise See picture Table 6 in Appendix
	CEN	Dummy variable: 1 if Central region; 0 otherwise See picture Table 6 in Appendix
	ALE	Dummy variable: 1 if Alentejo; 0 otherwise See picture Table 6 in Appendix
	ALG	Dummy variable: 1 if Algarve; 0 otherwise See picture Table 6 in Appendix
	LTV	Lisbon and Tagus Valley region Reference category for health administrative region
Unit type	USF-A	Dummy variable: 1 if USF without performance-based incentives; 0 otherwise
	USF-B	Dummy variable: 1 if USF with performance-based incentives; 0 otherwise
	USCP	Dummy variable: 1 if UCSP, former primary health care unit; 0 otherwise
	other units	Other types of primary health care units, e.g. UCC, USP and URAP. Reference category for unit type

The existing strong correlation between age and professional experience (r = 0.84) forced us to only use one of these variables, due to multicollinearity in a regression. We kept variable age as it is also a proxy for the years of professional experience.

### Empirical Model

Two equations were estimated, one for SAH and the other for burnout as follows:

$${\text{SAH - model:}}\,SAH_{i} = constant + \beta \,independent\,variables_{i} + {\upvarepsilon }_{{\text{i}}} ,$$

$${\text{Burnout - model}}:\,Burnout_{i} = constant + \delta \,independent\,variables_{i} + {\upmu }_{{\text{i}}} .$$

where β and δ correspond to a vector of the estimated coefficients for the independent variables, ε_i_ and μ_i_ is the residual term, and i represents an individual.

The set of independent variables in SAH-model could have included the burnout indicator and the Burnout-model could also have included SAH variable [46, 51]. However, this modelling specification raises the problem of endogeneity, resulting in biased and inconsistent coefficient estimates.

This means that there are unobservable variables which are correlated with both dependent and independent variables, such as lifestyle, family and personal life, spirituality, and genetic characteristics. Another possibility to explain endogeneity is by reverse causality, when the dependent variable has a causal effect on the independent variable, e.g. in the SAH-model, SAH may also determine burnout level.

One way to overcome the endogeneity problem would be to use instrumental variables. However, the variables available in our questionnaire are very limited, and so the use of instrumental variables is not an analytical option. Another possible alternative was to estimate simultaneous equations. However, burnout level and health status do not occur simultaneously. Also, the direction of the causality between health and burnout is not yet well established in the literature [49–51].

The main hypothesis to be tested by this research paper is whether professionals' well-being, measured by SAH and burnout, is influenced by 'place', which includes the type of healthcare unit, level of urbanization and administrative region. Since it was not our purpose to establish a causal relationship between health and burnout, and the estimated models are not trivial (both models are expressed in different forms of regression), we estimated two single equations for each well-being variable, one for SAH and another for burnout.

### Quantitative analysis

We began by presenting some descriptive statistics about the sample of primary healthcare professionals. Next, we estimated the SAH-model and the Burnout-model. Based on the nature of the dependent variable, an ordered probit model was estimated for self-assessed health, and a tobit model was estimated for burnout level. Estimations were performed for all the primary healthcare professionals, and for GPs and nurses separately. These estimations were not worth reporting for the technicians because of the relatively small sample for these professionals and the consequent absence of statistical significance of most results.

The models were estimated for two specifications, a reduced and a full model. The 'reduced model' included only independent variables related to ‘place’, that is, type of healthcare unit, urbanization density where the unit is located, and health administrative region. The 'full model' considered all independent variables described above and the potential relation between SAH and burnout levels for the set of all professionals. For this purpose, we have included the burnout indicator in SAH-model and the SAH variable in the Burnout-model. These estimations, as explained above, may be subject to endogeneity, so they may be taken here as a sensitivity analysis. The results are presented in the Appendix.

All estimations were done using the econometric software package STATA 15.

## Results

From the 19,486 existing primary healthcare professionals, 9,094 answered our questionnaire, corresponding to a 46.7% response rate. Splitting by professionals, we obtained the following response rates: 37.2% for physicians, 48.3% for nurses, and 52.6% of technicians.

### Descriptive statistics

The descriptive statistics concerning independent variables are displayed in Table 3.

**Table 3:** Baseline descriptive characteristics of independent variables

Variable		Total	GPs	Nurses	Technicians
Sample	Healthcare professionals, N	9,079	2,162	3,688	533
	USF-A, %	21.7	29.4	19.6	0.0
	USF-B, %	29.7	39.2	28.0	0.0
	UCSP, %	28.5	26.0	26.7	11.1
	other PHU units, %	20.2	5.4	25.7	88.1
Socio-economic	female, %	81.3	64.7	88.4	84.4
	age, mean ± st. deviation	46.4 ± 9.8	48.9 ± 12.6	43.5 ± 7.7	44.0 ± 8.3
	married, %	76.5	64.3	69.3	59.3
	nb_children, mean	1.3	1.3	1.4	1.2
	education, mean	16.5	16.6	16.4	16.4
	income—insufficiency %	36.1	14.1	40.8	50.8
Professional and labour	experience years, mean	21.3	22.7	20.6	19.7
	tenure contract,	80.3	80.1	82.4	75.4
Health status	SAH good and very good, %	71.4	74.5	71.9	67.7
	burnout, mean	41.8	46.8	37.2	36.4
Job satisfaction	OJS, mean	0.64	67.8	66.4	59.7

The average number of completed years of education is 16.5, and so it may be said that on average professionals have college education, as it would be expected. We also evidenced that burnout indicator was normally distributed.

The correlation between burnout indicator and SAH was not high, equal to **-**0.395, corresponding to about 16% of explanation.

The values of the burnout indicator were, on average, different between GPs and nurses. While nurses reported an overall burnout equal to 37.2, GPs reported a higher value equal to 46.8. On the other hand, nurses reported more often lower levels of health (SAH) than GPs. For instance, 28.1% of nurses reported self-assessed health between very poor and reasonable levels, while 25.5% of GPs reported the same levels. It is worth to highlight that the average age of nurses was 43.5 and of GPs was 48.9 years old. Besides only 11% of nurses and 35% of GPs were men.

### Reduced model

The results obtained in the reduced model are presented in Tables 6 (in the Appendix). At first glance, these results showed that burnout is a phenomenon emerging both at unit (both types of USF) and regional levels (especially in Central and Alentejo regions), while good (or poor) health tends to be locally observed at unit type. The level of urbanization seems not to have a relationship neither with health nor with burnout.

### Full model

#### Self-assessed health results

The results obtained for the self-assessed health full model are shown in Table 4.

**Table 4:** Results for full self-assessed health-model

		**ALL**		**GP**		**NURSE**	
		Coef	P > z	Coef	P > z	Coef	P > z
Socio-economic	male	0.074	0.070	-0.040	0.489	0.210	0.001
	age	-0.020	0.000	-0.020	0.000	-0.022	0.000
	married	-0.041	0.302	0.040	0.535	-0.076	0.169
	nb_children	0.056	0.004	0.045	0.143	0.049	0.069
	education	0.047	0.003	0.053	0.028	0.026	0.268
	income	0.313	0.000	0.331	0.000	0.294	0.000
Professional and labour	GP	-0.174	0.019				
	nurse	-0.220	0.001				
	tenure	0.032	0.444	-0.009	0.892	0.043	0.448
	leader	0.110	0.007	0.174	0.004	0.066	0.269
Job Satisfaction	OJS	0.017	0.000	0.018	0.000	0.017	0.000
Geographic	urban	0.057	0.227	0.084	0.328	0.021	0.724
	rural	0.006	0.955	0.025	0.885	-0.021	0.873
	NOR	-0.120	0.116	-0.314	0.030	-0.011	0.918
	CEN	-0.141	0.082	-0.326	0.033	-0.005	0.962
	LTV	-0.105	0.186	-0.348	0.019	0.057	0.606
	ALE	-0.201	0.033	-0.344	0.063	-0.052	0.679
Unit type	USF-A	0.148	0.006	0.177	0.183	0.188	0.003
	USF-B	0.142	0.006	0.238	0.070	0.118	0.054
	UCSP	0.164	0.002	0.314	0.020	0.116	0.071
Nb of obs		5,017		1,751		2,845	
LR chi2(18)		566.85		233.44		297.26	
Prob > chi2		0.000		0.000		0.000	
Pseudo R2		0.053		0.061		0.05	
Log likelihood		-5,069.396		-1,784.765		-2,856.997	

In general, and considering all health care professionals, SAH showed similar scores in all units. However, it was slightly higher in UCSP than in other units. Also, there were no significant differences across the various types of urbanization areas. Alentejo region had a significantly lower level of reported health status than Algarve. On the other hand, nurses reported somewhat lower levels of health than GPs and technicians tended to report better health status.

Also, individual characteristics provided predictors of SAH. Those who were leader, higher educated, with children, reporting sufficient income for family needs or reporting job satisfaction were more likely to report higher levels of health, while older people tended to report lower levels of SAH.

On the other hand, when comparing GPs and nurses, results showed that SAH was reported differently according to the type of healthcare unit organization. GPs in UCSP reported higher health status than those in other units, and nurses in USF-A reported better health than those in other units. Despite the small differences of health across regions, GPs in Algarve reported higher scores while in LVT and Central reported lower health condition. There was no significant regional difference for SAH reported by nurses.

There were common determinants of SAH between GPs and nurses. Younger individuals, with a higher level of professional satisfaction and considering their income as sufficient to their needs, tended to assess better scores to their health.

#### Burnout results

The estimated results for the full burnout model are presented in Table 5.

**Table 5:** Results for full burnout-model

		**ALL**		**GP**		**NURSE**	
		Coef	P > z	Coef	P > z	Coef	P > z
Socio-economic	male	-2.453	0.000	-3.260	0.000	-1.435	0.102
	age	-0.156	0.000	-0.173	0.000	-0.089	0.029
	married	0.971	0.089	1.494	0.147	0.553	0.455
	nb_children	-1.140	0.000	-1.614	0.001	-0.973	0.008
	education	-0.433	0.053	-0.846	0.025	-0.271	0.389
	income	-5.229	0.000	-7.929	0.000	-4.608	0.000
Professional and labour	GP	15.237	0.000				
	nurse	2.958	0.002				
	tenure	0.749	0.205	1.252	0.245	0.476	0.531
	leader	0.620	0.286	-1.246	0.185	1.867	0.020
Job Satisfaction	OJS	-0.411	0.000	-0.437	0.000	-0.400	0.000
Geographic	urban	-1.477	0.028	-2.693	0.047	-0.801	0.323
	rural	-1.066	0.463	0.110	0.967	-1.647	0.360
	NOR	3.493	0.001	8.086	0.000	2.358	0.097
	CEN	0.410	0.726	4.749	0.048	-1.315	0.384
	LTV	3.468	0.002	8.256	0.000	2.217	0.137
	ALE	2.439	0.072	7.046	0.016	1.209	0.475
Unit type	USF-A	1.302	0.088	6.464	0.002	0.317	0.710
	USF-B	0.878	0.235	5.362	0.010	0.792	0.336
	UCSP	-1.883	0.013	2.440	0.253	-1.993	0.021
_cons		77.387	0.000	97.310	0.000	74.615	0.000
Nb of obs		5,018		1,751		2,846	
LR chi2(18)		1,238.45		376.86		503.20	
Prob > chi2		0.000		0.000		0	
Pseudo R2		0.029		0.025		0.021	
Log likelihood		-20,991.808		-7,486.829		-11,728.554	

In general, burnout level was lower in UCSP than in other units and higher in USF-A. It was lower in urban areas than in moderately urban ones, but it was higher in both the North region and in Lisbon and Tagus Valley. GPs also reported higher levels of burnout, followed by nurses; while technicians reported lower levels of burnout.

Individual drivers of higher burnout included being female, younger, with few or no children, insufficiency of income to meet family needs and lower job satisfaction.

Across the different professional categories, GPs reported a higher level of burnout in USF-A and USF-B than in other units; nurses in UCSP reported lower levels of burnout than in other units. There was also a regional effect of burnout for GPs. In all regions, GPs had a higher burnout level than in Algarve. Such regional effect was not evident for nurses, however. Only in the North region, we found a slightly significant higher level of burnout.

In general, individual drivers for burnout were similar comparing GPs’ and nurses’ models. In both professional categories, older individuals, with one or more children, with sufficient income and job satisfaction, tended to present lower levels of burnout. The difference in burnout between GPs and nurses only lied on gender and leader effects. While being a male GP decreased the odds of suffering from a high level of burnout, being a leader nurse increased those odds.

In Appendix, Table 7, presents the results for these models including both SAH and burnout indicators as simultaneous independent variables. Comparing the results across Tables 4, 5 and Table 7 in Appendix there are no significant differences worth to remark. Results, in general, show the same significance and sign for the estimated coefficient. The additional information obtained in Table 7 in Appendix is the inverse relationship between SAH and burnout levels, as expected.

Also, in the Appendix, the results for the different dimensions of burnout are presented. Tables 8, 9 and 10 in Appendix display the results for the full model applied to the burnout dimensions: personal, work-related, and patient-related. These results are presented for all professionals in the sample, and separately for GPs and nurses. A general first observation makes it clear that GPs had higher levels of burnout across health units and regions, but urbanization may mitigate these levels of burnout. Burnout felt by nurses seems to be less sensitive to urbanization and regional effects.

## Discussion

### Summary of findings

The well-being of professionals has fundamental importance to guarantee that health systems can pursue their main goal of improving people’s health. The current analysis aimed to determine individual drivers for well-being, measured by self-assessed health and by burnout, to verify whether there are ‘place’ determinants of well-being and whether there are differences between GPs and nurses.

The main results show that, firstly, nurses generally report slightly worse health than GPs, though suffering lower levels of burnout; secondly, 'place' effects are more significant in GPs than in nurses.

### Self-assessed health

Concerning self-assessed health (SAH), we found that GPs tended to report higher health status in USF-B and in UCSP units; on the other hand, in Algarve we evidenced higher scores. This region has pleasant climate and living conditions, which may contribute to a better reported SAH. Moreover, despite seasonal demographic fluctuations, these do not affect the demand for primary care of the national health service. The worst SAH was reported in Alentejo, where there is the highest suicide rate, and the lowest demographic density. These features may explain the no 'urbanization effect'. For nurses, in general, no significant 'place' effects were found in SAH.

### Burnout levels

Concerning burnout, results showed that GPs give higher scores in USF-A and USF-B. In addition, burnout levels were higher in all regions when compared to Algarve, and the most intense levels of burnout are found in North and Lisbon and Tagus Valley. These results did not support previous evidence from Maroco et al. [39] who found that burnout syndrome among physicians was uniform across Portugal mainland regions. However, their sample [39] included physicians from different health care settings while our sample includes only primary healthcare physicians. Also, the burnout measurement scale was different. This may be a reason for the existing disparity of the results.

We also found that for GPs, 'urbanization effect' tended to decrease burnout intensity. This is different from what was seen by Saijo et al. [34], but identical to Lavanchy et al. [35]. The majority of the cities in Portugal have a medium-small size and provide a set of services and social environment, which are useful for personal and family life without heavy levels of pollution, traffic or crime. In general, the quality of life in Portuguese cities is good and people rather live and/or work in urban areas than in rural ones or outskirts/semi-urban.

We found 'place effects' for nurses in UCSP, where burnout levels tend to be low. Nurses in the Northern region suffer higher levels of burnout. This result is partially identical to the one found by the previous work [39] where it was identified higher burnout levels in the North and Centre of Portugal.

There was a tendency for nurses and GPs to suffer higher burnout in the Northern region and for GPs in Lisbon and Tagus Valley. This tendency may be related to the measures and policies followed by Regional Health Authorities. Good governance and organization measures may lead to the development of work quality. Additionally, weak leadership traits, poor staff management, and resource inadequacy of some of the Groups of Primary Care Centers do not boost work satisfaction. Therefore, these weak organizational and leadership attributes trigger burnout.

The case of GPs in Lisbon and Tagus Valley, where they tended to present higher levels of burnout, was also expected. In this region, the number of people not enrolled in a GP list is very large, due to the demographic pressure not timely addressed by the authorities, and by the consequent higher demand for consultations. Another fact that may explain this result comes from the tension arising from the lower levels of patient satisfaction in Lisbon and Tagus Valley [46], mainly caused by the difficult access to healthcare services [46].

Finally, in USF-B, GPs tended to report lower health status and higher levels of burnout, but this was not the case for nurses. One possible explanation for this result is related to the type of organization and contracting mechanisms of these units, characterized by some level of autonomy, participative management and pay for performance incentives to GPs. It may be the case that the pressure from this type of organization is causing chronic stress, anxiety, depression, and burnout feelings.

### Individual drivers of well-being

Concerning the individual drivers of well-being, our analysis showed that there are common determinants of SAH and burnout level between GPs and nurses. In the case of SAH, we found that being older is correlated with lower levels of health, as expected from the conceptual model of health demand [52] while being professionally more satisfied and reporting sufficient income to face family needs contribute to better health status.

In the case of burnout, the common determinants of lower burnout intensity between GPs and nurses included being older, with children, with a sufficient household income, and being more satisfied with the job. Among the socioeconomic factors, age tended to have a significant relationship with burnout. As found in most studies, younger individuals tended to experience stronger symptoms of burnout [25, 26, 38, 53]. Although this relationship may not be linear and so, higher levels of burnout in older ages may be found by other researchers [54].

Concerning the role of the sufficiency of the income for family needs, our findings suggest that higher income tended to be related with better health status and with lower burnout levels, both for GPs and nurses [26]. Several authors have identified a relationship between better payment or income earned and higher job satisfaction, which in turn decreases burnout level [54–56]. Despite this general view, Picquendar et al. [53] did not find such relation between payment and burnout syndrome and Linzer et al. [57] concluded that the relevant factor for physicians was rather the relationship with patients than the monetary compensation. The income variable used in our work, in fact, measures better the family well-being concern than the absolute value of income or the implicit relationship with job satisfaction. In a family-based society, our results are expected to be found.

Concerning job satisfaction, and as expected from previous work [25, 26, 58–61], we found that higher levels of satisfaction were correlated with better health status and lower burnout. In fact, job satisfaction contributes to the mitigation of the set of burnout dimensions, including lower emotional exhaustion and depersonalization. Simultaneously, job satisfaction is a consequence of good human resource policy (good leadership and communication), high levels of morale, good resources, as well as favourable attitudes towards the functioning and continuous quality improvement of the unit. These job satisfaction features contribute to a sense of personal and professional accomplishment.

### Differences between nurses and GPs and between genders

The proportion of men and women in primary health care is unequal and the majority of professionals are women, about 90% of nurses and 65% of GPs. However, it can be noticed from other statistics [62–64] that the percentage of males in leader positions is higher that the percentage of males in the working place.

We found that nurses reported a slightly worst health than GPs, but these tended to suffer from higher levels of burnout, unlikely previous findings [38]. Male gender was correlated with better health, among nurses, and lower burnout intensity, among GPs. So, being male seems to be protective of lower health and from higher burnout. Our result is not coincident to the one discussed before [25], where it was stated that in Southern European countries, male GPs tended to have a higher level of burnout. The existing ratio between nurses and physicians may explain our finding. On the other hand, as the share of male leaders (about 23.8% of the sample) is larger than the share of males in workplace (about 19% of the sample) there is some male style of leadership which favours the smaller share of males in the workplace. This may be observed in the assessment professionals do about the human resources policy and the coordination of the health in the sample unit [46, 63]. On average, males tend to be more satisfied with these specific aspects of the workplace than females. These differences may contribute to the differences found between genders. Finally, concerning self-assessed health, women tend to report worst health than men. But under the control of risk factors and socioeconomic factors, men and women may have identical health. In fact, this comparison may not be so clear [65, 66]**.**

### Study limitations and strengths

Well-being is measured by self-reported variables, which may yield to a self-reported bias [63]. However, due to the very large sample used, there is a trade-off between positive and negative biases, which may result in a strong mitigation of this bias.

Another limitation of our work is the possible bias of respondents’ decision not to fill the questionnaires. However, 46.4% response rate yields a large sample size of 9,094 professionals, a significant sample of all primary healthcare professionals of mainland Portugal. On the other hand, analysing the sociodemographic characteristics we confirmed that the obtained sample is representative of the workforce employed in the primary health care in mainland Portugal. Therefore, we tend to minimize this possible constraint.

The major strength of this analysis is the expansion of understanding of the relationship between well-being and ‘place’, defined by the type of health unit, the urbanization and the region. We not only contribute to the discussion on the relationship between well-being and the level of urbanization, but we also add knowledge about the relationship between well-being and the type of primary health care units and the Portuguese regions. The second major strength is the use of data from a very large sample of primary health care professionals. Finally, this analysis also includes simultaneously two important concepts related with well-being of professionals, which is not very common in the literature.

### Policy recommendations

The analysis presented here provides a set of policy recommendations, which may contribute to the final aim of improving the workforce and, indirectly, the quality of the health care provided. Several measures at the health care unit and regional level should be considered to reduce GPs work stress and consequent burnout. Improving job satisfaction contributes not only to the reduction of burnout, but also to the improvement of the health of professionals. Job satisfaction is improved with a better communication and leadership from the top organization structures, also enhanced with morale in the workplace, and better resources to provide care. Other measures on health resources organization such as less bureaucracy to be attended, more time to provide personalised care, supportive supervision and coordination, enhancement of teamwork, mechanisms to hold professionals accountable for their actions, and non-pecuniary incentives may be considered to improve job satisfaction. Among the several factors that influence health status and burnout levels, income has a clear far-reaching effect, and so it may be considered in medium run policies by the Ministry of Health.

## Conclusions

This work has estimated and described the main individual drivers of health and burnout for GP and nurses. The well-being of these professionals is fundamental to ensure that health systems can keep improving people’s health. It was suggested that policy measures aiming the improvement of job satisfaction, either at the health care unit level and regional level, could contribute to the improvement of professional well-being.

## Data Availability

The datasets used during the current study cannot be made available as stated in the funding contract with the Ministry of Health of Portugal.

## Appendix

**Table 6:** Results for Self-Assessed Health and Burnout models—reduced models

		SAH-model		Burnout-model	
		ordered probit		tobit regression	
		Coef	P > z	Coef	P > z
Geographic	urban	0.009	0.828	0.055	0.936
	rural	-0.135	0.139	1.352	0.363
	NOR	0.083	0.222	0.112	0.919
	CEN	0.013	0.862	-2.417	0.042
	LTV	0.020	0.782	2.018	0.083
	ALE	0.000	0.996	-2.724	0.049
Unit type	USF-A	0.148	0.001	6.408	0.000
	USF-B	0.262	0.000	2.321	0.000
	UCSP	-0.050	0.229	5.334	0.000
_cons				36.962	0.000
Nb of obs		5989		6070	
LR chi2(18)		102.98		150.45	
Prob > chi2		0.0000		0.0000	
Pseudo R2		0.0080		0.0029	
Log likelihood		-6422.7937		-26,106.38	

**Table 7:** Results for Self-Assessed Health and Burnout models for all professionals

		SAH-model		Burnout-model	
		Coef	P > z	Coef	P > z
Socio-economic	male	0.015	0.711	-2.087	0.000
	age	-0.027	0.000	-0.261	0.000
	married	-0.018	0.653	0.755	0.156
	nb_children	0.031	0.115	-0.847	0.001
	education	0.040	0.012	-0.191	0.361
	income	0.198	0.000	-3.597	0.000
Professional and labour	GP	0.229	0.003	14.301	0.000
	nurse	-0.158	0.017	1.813	0.039
	tenure	0.055	0.186	0.905	0.101
	leader	0.137	0.001	1.197	0.027
Job Satisfaction	OJS	0.007	0.000	-0.323	0.000
Geographic	urban	0.024	0.622	-1.174	0.062
	rural	-0.021	0.840	-0.996	0.463
	NOR	-0.037	0.635	2.861	0.005
	CEN	-0.143	0.083	-0.320	0.769
	LTV	-0.020	0.805	2.920	0.006
	ALE	-0.155	0.107	1.385	0.274
Unit type	USF-A	0.196	0.000	2.076	0.004
	USF-B	0.178	0.001	1.602	0.020
	UCSP	0.129	0.016	-1.037	0.141
Well-being variables	SAH			-8.539	0.000
	Burnout	-0.027	0.000		
_cons				104.634	0.000
Nb of obs		5,017		5,017	
LR chi2(18)		1257.210		1931.650	
Prob > chi2		0.000		0.000	
Pseudo R2		0.117		0.045	
Log likelihood		-4724.215		-20,641.378	

**Table 8:** Results Burnout model—Burnout dimensions for all professionals

Burnout dimensions		Personal-related		Work-related		Patient-related	
		Coef	P > z	Coef	P > z	Coef	P > z
Socio-economic	male	-4.916	0.000	-2.347	0.000	-0.245	0.725
	age	-0.135	0.000	-0.194	0.000	-0.148	0.000
	married	1.212	0.068	0.647	0.273	1.118	0.099
	nb_children	-1.107	0.001	-0.976	0.001	-1.353	0.000
	education	-0.256	0.326	-0.261	0.261	-0.823	0.002
	income	-6.079	0.000	-5.622	0.000	-4.272	0.000
Professional and labour	GP	14.693	0.000	14.466	0.000	17.321	0.000
	nurse	2.766	0.011	3.208	0.001	3.258	0.004
	tenure	1.041	0.130	0.661	0.281	0.530	0.450
	leader	0.972	0.150	1.078	0.073	-0.176	0.799
Job Satisfaction	OJS	-0.456	0.000	-0.447	0.000	-0.352	0.000
Geographic	urban	-1.837	0.019	-1.705	0.015	-1.230	0.124
	rural	-1.597	0.345	-1.102	0.464	-0.760	0.660
	NOR	4.686	0.000	3.679	0.001	2.311	0.077
	CEN	2.298	0.092	0.352	0.771	-1.260	0.365
	LTV	5.243	0.000	4.151	0.000	1.298	0.339
	ALE	4.013	0.011	3.253	0.021	0.279	0.862
Unit type	USF-A	-0.741	0.404	-0.558	0.480	5.450	0.000
	USF-B	-1.133	0.188	-1.265	0.099	5.408	0.000
	UCSP	-4.178	0.000	-3.658	0.000	2.236	0.013
_cons		79.422	0.000	82.836	0.000	71.718	0.000
Nb of obs		5018		5018		5018	
LR chi2(18)		1030.09		1210.69		1029.29	
Prob > chi2		0.000		0.000		0.000	
Pseudo R2		0.023		0.028		0.024	
Log likelihood		-21,457.36		-21,122.62		-21,226.267	

**Table 9:** Results Burnout model—Burnout dimensions for GP

Burnout dimensions		Personal-related		Work-related		Patient-related	
		Coef	P > z	Coef	P > z	Coef	P > z
Socio-economic	male	-4.766	0.000	-2.817	0.003	-2.396	0.021
	age	-0.179	0.000	-0.217	0.000	-0.126	0.004
	married	1.389	0.239	0.898	0.392	2.475	0.034
	nb_children	-1.529	0.006	-1.687	0.001	-1.657	0.003
	education	-0.985	0.023	-0.745	0.053	-0.863	0.044
	income	-8.547	0.000	-7.964	0.000	-7.526	0.000
Professional and labour	tenure	1.702	0.167	1.491	0.174	0.608	0.618
	leader	-1.436	0.182	-0.802	0.401	-1.499	0.159
Job Satisfaction	OJS	-0.464	0.000	-0.465	0.000	-0.410	0.000
Geographic	urban	-3.502	0.024	-2.462	0.075	-2.488	0.106
	rural	-0.385	0.901	0.596	0.828	0.036	0.990
	NOR	8.421	0.001	6.747	0.004	9.403	0.000
	CEN	4.695	0.089	3.485	0.154	6.390	0.019
	LTV	9.235	0.001	7.538	0.001	8.491	0.001
	ALE	7.114	0.033	5.936	0.045	8.580	0.009
Unit type	USF-A	2.709	0.260	4.911	0.022	11.854	0.000
	USF-B	1.742	0.465	3.654	0.085	10.928	0.000
	UCSP	-1.597	0.513	0.914	0.674	7.908	0.001
_cons		106.794	0.000	103.446	0.000	84.426	0.000
Nb of obs		1751		1751		1751	
LR chi2(18)		335.72		407.24		269.28	
Prob > chi2		0.000		0.000		0.000	
Pseudo R2		0.0216		0.0264		0.0175	
Log likelihood		-7,600.705		-7,502.344		-7,564.115	

**Table 10:** Results Burnout model—Burnout dimensions for nurses

Burnout dimensions		Personal-related		Work-related		Patient-related	
		Coef	P > z	Coef	P > z	Coef	P > z
Socio-economic	male	-5,001	0,000	-1,475	0,108	1,971	0,064
	age	-0,034	0,476	-0,116	0,006	-0,130	0,008
	married	1,092	0,209	0,491	0,526	0,001	0,999
	nb_children	-0,901	0,036	-0,547	0,153	-1,484	0,001
	education	0,051	0,890	-0,067	0,840	-0,802	0,036
	income	-5,337	0,000	-5,091	0,000	-3,635	0,000
Professional and labour	tenure	0,739	0,408	0,101	0,899	0,576	0,532
	leader	2,600	0,006	2,385	0,005	0,617	0,527
Job Satisfaction	OJS	-0,450	0,000	-0,443	0,000	-0,326	0,000
Geographic	urban	-0,885	0,353	-1,307	0,124	-0,496	0,613
	rural	-2,049	0,332	-2,185	0,246	-1,124	0,606
	NOR	3,463	0,038	2,901	0,051	0,927	0,590
	CEN	0,818	0,645	-1,198	0,449	-3,424	0,061
	LTV	3,786	0,031	3,208	0,040	-0,102	0,955
	ALE	2,720	0,171	2,491	0,160	-1,463	0,476
Unit type	USF-A	-1,283	0,200	-1,515	0,090	4,007	0,000
	USF-B	-0,963	0,319	-1,380	0,110	5,051	0,000
	UCSP	-3,686	0,000	-3,792	0,000	1,561	0,137
_cons		72,129	0,000	79,374	0,000	73,886	0,000
Nb of obs		2846		2846		2846	
LR chi2(18)		480,350		567,060		296,180	
Prob > chi2		0,000		0,000		0,000	
Pseudo R2		0,020		0,023		0,012	
Log likelihood		-12,043.384		-11,829,958		-11,890,948	

## Abbreviations

GP: General practitioner
SAH: Self-assessed health
OJS: Overall job satisfaction
USF-A: Family Health Units type A
USF-B: Family Health Units type B
UCSP: Traditional primary health care units
NOR: North Region
CEN: Central Region
LVT: Lisbon and Tagus Valley Region
ALE: Alentejo Region
